# Neurosurgery for Optic Pathway Glioma: Optimizing Multidisciplinary Management

**DOI:** 10.3389/fsurg.2022.884250

**Published:** 2022-05-04

**Authors:** Derek C. Samples, Jean M. Mulcahy Levy, Todd C. Hankinson

**Affiliations:** ^1^ Department of Neurosurgery, Children’s Hospital Colorado, Aurora, CO, United States; ^2^ Department of Pediatrics (Center for Cancer and Blood Disorders), University of Colorado School of Medicine and Children’s Hospital Colorado, Aurora, CO, United States; ^3^ Morgan Adams Foundation Pediatric Brain Tumor Research Program, University of Colorado School of Medicine and Children’s Hospital Colorado, Aurora, CO, United States

**Keywords:** optic pathway glioma, pediatric neurosurgery, neurofibromatosis (NF), BRAF, biopsy

## Abstract

Optic pathway glioma (OPG) comprises 10% of pediatric brain tumors and 40% of all pediatric low-grade gliomas (pLGGs). While generally considered benign pathologically, many require interventions with chemotherapy, radiation, or targeted therapies. Management has historically foregone tissue diagnosis given the classical clinical/radiographic presentation of these tumors, inability to safely remove the lesions surgically, and efficacy and safety of available chemotherapy options. Furthermore, when considering such aspects as their delicate location, the role of surgery continues to be heavily debated. More recently, however, a greater understanding of the genetic drivers of OPGs has made operative tissue sampling a critical step in management planning, specifically for patients without Neurofibromatosis, Type I (NF1). Given the need for long-term, complex management of pediatric OPGs, it is crucial that a multidisciplinary approach is employed, and the rapidly expanding role of molecular characterization be incorporated into their management.

## Background

Pediatric low-grade glioma (pLGG) is the most common CNS tumor in children, constituting 40%–50% of all pediatric brain tumors (1). While they can be found in a multitude of locations, pLGGs arise in the optic pathway/hypothalamic region about 40% of the time (2, 3). Optic pathway glioma (OPG) can arise in the optics nerve(s), optic chiasm, optic tract, lateral geniculate body, and hypothalamus and is most typical in the chiasmatic/hypothalamic region (4, 5). OPGs are diagnosed by the first decade of life in 75% and the second decade of life in 90% of patients (6). They can be sporadic or in association with Neurofibromatosis Type 1 (NF1). Approximately 15%–20% of patients with NF1 will develop OPGs and up to 70% of pediatric OPGs are diagnosed in NF1 patients (4, 7–11). Compared to sporadic OPGs (**Table 1**), which can present throughout childhood, NF1-associated OPGs typically arise by 5 to 6 years of age (9). Presentation results from involvement in or compression of the optic pathway and diencephalon, CSF obstruction from hydrocephalus, or mass effect from large tumors (8). Children may exhibit clinical signs of visual impairment, endocrine dysfunction, diencephalic syndrome (Russell syndrome), and life-threatening intracranial hypertension (2, 5, 6, 8).

**Table 1:** Characteristics of OPG is NF1 and non-NF1 patients.

NF1 patients	Non-NF1 patients
Earlier age of presentation (age 3–6 years)	Can present anywhere throughout childhood
Tumor location tends to be more anterior and potentially involve only the nerve	Tumor location can be more posterior
50%–70% aysmptomatic	More likely to cause clinical symptoms/visual impairment
NF1 favorable prognostic marker	BRAF-KIAA fusion is favorable prognostic marker
Less likely to progress	More likely to progress
Only approx. 35% require treatment	>90% of patients will require treatment

Histologically, these tumors are low-grade with majority being Pilocytic Astrocytomas (PA) (5, 7, 8). However, pilomyxoid astrocytoma (PMA), oligodendroglioma, and ganglioglioma have also been confirmed on pathology (5, 8, 11, 12). While progression to high grade malignancy is uncommon, compared to other pLGGs, OPGs have a higher tendency to progress, representing 50% of pLGGs that undergo dissemination (11). More aggressive tumors are observed in females, locations posterior to the optic chiasm, tumors with pilomyxoid features, and in younger (<2 years) or older (>10 years) children (3, 5, 13, 14). Symptomatic presentation was strongly predictive of future deterioration (OR 14.8) compared to incidental tumors (11). The presence of NF1 has been demonstrated to be a favorable prognostic maker (2, 5). Patients with NF1-associated OPG have less increase in intracranial pressure (ICP), less decrease in vision, and fewer fundi abnormalities. Radiographic progression, visual deterioration, and endocrine damage are also less frequent in this population (11).

The clinical course of OPG may be unpredictable due to variability in the tumor’s natural history. The behavior of these tumors, while commonly thought quite indolent, can include progression, stable disease, and, on occasion, regression (3, 8, 15). Tumor quiescence (a state of reversible cell cycle arrest) has been observed in OPG. Particularly, in NF1 patients, these tumors are less common to progress compared to their sporadic counterparts (10, 11). It has been reported that up to 50% of OPGs will remain stable but a significant number will progress to require treatment, or progress after treatment (8). The inherent heterogeneity of this condition with an erratic natural history that can span childhood highlights the importance of long-term multidisciplinary management.

Management of OPGs is based on patient age, clinical presentation, location, surgical resectability, and, when available, histopathological findings. Treatment options include observation, chemotherapy, surgical biopsy, resection, radiotherapy, and molecularly targeted therapy. Despite the understanding that a significant portion of these tumors will remain stable, as many as 35% of OPGs in NF1 patients will require treatment at presentation and more than 90% of sporadic OPGs will require treatment (2, 11). Long-standing first line treatment is chemotherapy with Carboplatin and Vincristine (CV) (4, 5, 7, 8, 16). Alternatives therapies have included TPCV (thioguanine, procarbazine, lomustine, vincristine), vinblastine monotherapy, and more recently targeted therapies. VEGF inhibition is also being studied using bevacizumab containing regimens (i.e., NCT02840409: Vinblastine ± Bevacizumab in Children with Unresectable or Progressive Low Grade Glioma) (2, 5). Radiotherapy, while highly effective, is largely avoided due to the risk of secondary malignancies, neurotoxicity, endocrinopathies, and neuro cognitive decline (4). In the setting of NF1, where risks of malignancy with ionizing radiation are even higher (50%), greater emphasis is placed on up-front chemotherapy when indicated. Recurrent or progressive OPGs are treated with additional chemotherapy (Carboplatin or Vinblastine monotherapy or combination bevacizumab/irinotecan) (2). These decisions are often made without the benefit of genetic analysis to guide therapy choices.

Complete surgical resection of OPG is generally not possible due to their location and complex anatomical relationship to sensitive structures. It is important to note that various opinions exist regarding surgical strategies for OPG (3–6, 8, 12, 13, 17). Broadly accepted surgical indications include management of hydrocephalus with CSF diversion (most commonly VP shunt), debulking for symptomatic/life-threatening mass effect, and drainage of symptomatic cystic components (5, 6, 8, 11, 12, 18, 19). Regardless, no treatment modality has surpassed conventional chemotherapy as the gold standard for tumor control in pediatric OPG.

## Genetics of Optic Pathways Glioma

The role of tissue biopsy has been variable due to the surgical risk associated with the location of OPG as well generally favorable response to chemotherapy. Biopsy rates have increased over recent years with the emergence of targeted therapies and a greater understanding of the genetic drivers of pLGG. A more sophisticated understanding of the biological landscape of pLGGs, first evident in NF1 patients harboring PAs, has refocused treatment of OPGs on molecularly targeted options. Compared to their adult counterparts, pLGGs exhibit a greater likelihood to activate BRAF, with subsequent upregulation of the Ras/MAP-Kinase pathway. In fact, extensive genetic analysis has discovered that they represent a single pathway disease with genetic alterations converging on the MAPK pathway (10, 20, 21). It is difficult to find exact numbers indicating the percentage of OPG with BRAF alterations due to the previous avoidance of biopsy in this patient population. One small study of patients with PA of the optic nerve found 1 of 13 patients (8%) was postive for BRAFV600E, 3 were positive for BRAF fusion (13%) and 5 were indeterminate (39%) (22). Another small study pediatric patients with NF1-like OPGs but no clinical NF1 diagnosis found 4 of 11 patients tests (36%) contained BRAFV600E, 3 (27%) had BRAF:KIAA fusions and 1 (9%) had a gain of function mutation in KRAS (23). These studies support the presece of MAP kinase pathway alteration in OPGs.

In NF1 patients, alteration of the neurofibromin 1 gene on chromosome 17 leads to dysregulation of neurofibromin activity. Neurofibromin works as a tumor suppressor by reducing RAS-GTP mediated activation of its effector pathways, including MAPK and phosphoinositide 3-kinase (PI3K), and thus regulates cell growth and proliferation though downstream proteins (e.g., RAS, BRAF, mTOR). Dysregulation of neurofibromin results in upregulation of Ras and mTOR activity, leading to a pro-mitotic state. In pLGG, abnormal MAPK activation is the most frequent biological aberration demonstrated, and OPGs also frequently exhibit mutations in the same pathway (2). The two most common alterations in pLGG (including OPG) are in the BRAF gene with either KIAA1549:BRAF fusion or point mutation of BRAFV600E. Unique fusions that activate MAPK have also been reported (24). Mutations or fusions involving the FGFR1 or NTRK families in pLGG are more recent findings. Recurring activating FGFR1 and NTRK1 alterations in non-cerebellar PA results in MAPK pathway activation (20) and these alterations have been identified in OPG (25–27).

Genetic anomalies in pLGG vary based on histology, with BRAF fusion seen more commonly in Pilocytic Astrocytoma (PA), while V600E mutation is more prevalent in pleomorphic xanthoastrocytoma (PXA) and ganglioglioma (10, 28). The latter is also found more often in supratentorial lesions. FGFR1 mutations are more typical of midline tumors (10). Such alterations can be excellent candidates for molecular targeted therapies. However, to leverage this biological information, tissue diagnosis and molecular classification is of utmost importance. Advocacy for standardization of molecular profiling is now being seen throughout the field (5, 10, 16, 29). Contemporary work has demonstrated the value of standardizing diagnostic processes by supplementing pathological diagnosis with DNA methylation profiles. DNA methylation profiling is highly robust and reproducible despite sample quality and, with respect to CNS tumors, has been shown to improve diagnostic accuracy when used in combination with histological and molecular tumor classification (30, 31). Additional evidence of the diagnostic value of this process is reflected in recent updates to the WHO classification of pediatric gliomas (32). As these standardized tumor classifiers become more widely utilized and refined their ability to enhance diagnostics, and therefore management, of such entities as the focus of the current work will be further appreciated.

## Implications of Molecular Characterization on Management

The goal of care in patients with OPG is to preserve neurological function and maximize quality of life. Based on the indolent nature of many OPGs and the success of current chemotherapy regimens, initial management may consist of close observation, with chemotherapy used in cases of visual deterioration or radiographic progression (7, 8, 11). In some cases, however, chemotherapy alone may not provide tumor control or visual improvement (33–36). In addition to the side effects that are often associated with cytotoxic chemotherapy (e.g., myelosuppression, neurotoxicity, hypersensitivity reactions), progression rate on chemotherapy can be as high as 50% (37). Although gross total resection of OPG may not often be possible, significant operative debulking has demonstrated satisfactory outcomes in selected cases, and there exist clear indications for other surgical interventions (See section on Surgical Nuances) (4–6, 8, 12, 13, 18, 19, 38). Illustrating the variable opinions regarding the role of surgery for OPG, a consensus statement from 2011 suggested limiting the role of tissue biopsy to sporadic OPGs in relevant clinical trials or those with atypical radiographic features, all in the setting of multidisciplinary discussion (3).

Given today’s level of knowledge regarding the biology of pLGG, the value of tissue diagnosis is a critical factor. Tissue characteristics that can only be revealed through direct sampling may guide clinical management. For example, OPGs possessing BRAF fusion have shown a tendency to arrest and are even prone to senescence (11, 39), and prolonged progression free survival has been associated with BRAF-KIAA fusion positive OPGs (37). Alternatively, V600E+ tumors have been shown to act more aggressively (29, 40). Historically, tumors with pilomyxoid features have also been associated with worse outcomes (14). While the prognostication of this disease based on molecular characterization continues to evolve, when considered in the setting of emerging targeted therapies, strong consideration for tissue diagnosis is warranted.

While prospective, randomized control trials are ongoing, success with molecular targeted therapy in OPG has been well documented. The first MEK inhibitor (MEKi) was developed in 1995 and targeted BRAF inhibitors started with sorafenib, a broad-spectrum kinase inhibitor, followed by vemurafenib and dabrafenib, BRAF V600E specific targeted inhibitors. The initial clinical experience with sorafenib demonstrated the importance of knowing the underlying genetic make-up of a tumor being treated with targeted therapy. A phase II trial of sorafenib in pLGG was closed early to an unexpectedly high rate of rapid and early progressive disease, in 9 of 11 patients enrolled. It was determined that sorafenib may lead to paradoxical ERK activation in NF1-deficient cells as well as BRAF wild-type and KIAA1549:BRAF fusion cells (41).

Since the early studies with sorafenib, the field has focused on more specific targeted therapy (**Table 2**). Dabrafenib and vemurafenib have been studied in the management of V600E+ pediatric gliomas. A phase I/IIa trial with dabrafenib in pLGG found a 1-year PFS of 85% (42). Early studies using Vemurafenib had similar promising results (43). MEK inhibition is also showing excellent clinical potential in pLGG with MAPK pathway alterations. Selumetinib, a second generation MEKi, yielded 96% PFS in NF1 patients with BRAF aberrations at 2 years, and stable to improved vision for patients with OPGs (44). In another phase II study, Fangusaro et al demonstrated improved PFS as well as visual function when treating recurrent/progressive OPGs (2). Selumetinib has been shown to be effective in non-NF pLGG with a 2-year PFS of 69% in recurrent/refractory pLGG (45). When studied specifically in recurrent non-NF OPG and hypothalamic pLGG, the 2-year PFS was 78%, with 21% of patients having improved and 68% with stable visual acuity (2). Other MEKi’s, such as trametinib and binimetinib, are being studied, and some used off-label for MAPK-activated pLGG with exciting results (29). Trametinib has been shown to be effective in treatment-refractory NF-1 related pLGG (46) and non-NF1 pLGG with other MAPK pathway alterations (47). Building on the success of single agent therapy, the combination of BRAF and MEK inhibition is also becoming a mainstay in the treatment of pediatric BRAF V600E tumors (48, 49).

**Table 2:** Summary of targeted therapies available for OPG treatment.

Targeted therapy	Molecular pathway	Molecular target(s)
Sorafenib	Raf/MEK/ERK	C-Raf, B-Raf, surface kinases
Vemurafenib	Raf/Mek/ERK	BRAF, BRAF-V600E
Dabrafenib	Raf/Mek/ERK	BRAF, BRAF-V600E
Selumetinib	Raf/Mek/ERK	MAPK1, MAPK2
Trametinib	Raf/Mek/ERK	MEK1, MEK2
Binimetinib	Raf/Mek/ERK	MEK1/2
Everolimus	PI3K/Akt/mTOR	mTOR
Larotrectinib	Raf/Mek/ERK	NTRK fusions
Bevacizumab	VEGF/Ras/mTOR/ERK angiogenesis	VEGF-A

*MEK, mitogen activated extracellular signal regulated kinase; ERK, extracellular signal regulated kinase; PI3K, Phosphoinositide 3-kinase; mTOR, mammalian target of rapamycin; VEGF, vascular endothelial growth factor; NTRK, Neurotrophic receptor kinase.*

Other potential therapies include inhibition of mTOR with everolimus, which has shown good results with radiographic response and tumor stabilization in a phase II trial in NF1 patients (11). Identification of FGFR1 and NTRK abnormalities will allow the use of FGFR1 and NTRK inhibitors in non-surgically accessible lesions. While no OPGs were included, a phase II study with larotrectinib, a TRK inhibitor, demonstrated good tolerance and encouraging activity in patients with tumors harboring NTRK fusion genes (50). FGFR specific inhibition is showing promise in early adult data (51), and is in clinical trials in pediatric patients through the Pediatric MATCH treatment Trial (NCT03210714). One of the struggles of treatment with targeted agents is it is currently unclear when to discontinue therapy. There is evidence of “rebound” tumor growth when patients stop targeted therapy, with additional evidence that restarting therapy can be effective (52). It is also unclear if intermittent dosing of MAP kinase inhibitors, allowing for “drug holidays”, is a potential therapy option. Evidence in pre-clinical melanoma models suggest that intermittent and continuous MAP kinase pathway inhibition (with mono or dual therapy) results in similar impact on tumor growth (53). Investigation into the use of intermittent dosing in LGG are ongoing (NCT04485559).

While some patients are deriving benefit from MAPK targeted therapy, some do not, suggesting innate resistance. A small but significant group of patients develop resistance to the drugs over time. Gaps in the current knowledge regarding the causes of innate and acquired resistance is in part the result of a lack of tissue sampling that resulted from historical practice patterns. Further detailed tissue study would help refine our understanding of established molecular vulnerability and potentially lead to the identification of new biological targets. Furthermore, the evolving re-classification and addition of new tumor types from the World Health Organization, including pediatric gliomas that are “MAPK pathway-altered”, exemplifies the value of molecular classification (9, 54).

**Figure 1:**
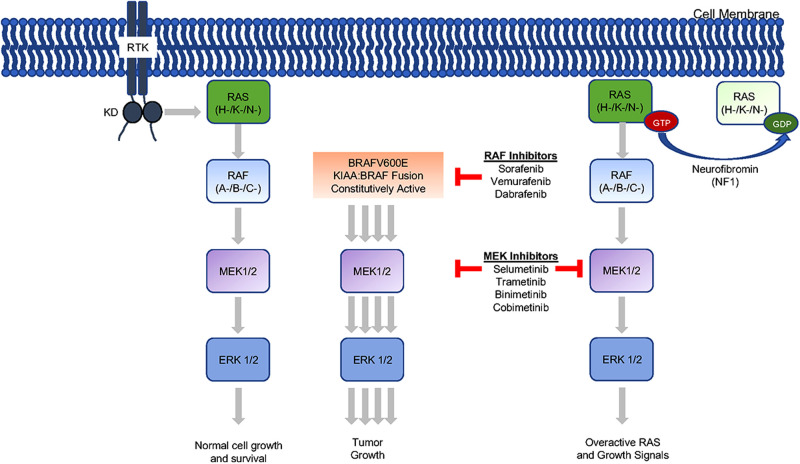
Mitogen-activated protein kinase (MAP kinase) pathway inhibition. The MAP kinase pathway can be inhibited at multiple sites with currently clinically available drugs. Of note, sorafenib (RAF inhibitor) was studies in the low-grade glioma population but found to result in unexpected progression in some patients (29). The additional drugs are use as monotherapy and in combination to treat low-grade gliomas.

Management goals in the era before targeted therapy were to preserve as much function as possible as well to maintain an acceptable quality of life. As stated above, this remains true, but increasingly individualized multidisciplinary management is now at the forefront of OPG treatment (28). In this regard, when compared to chemotherapy, which has sometimes shown suboptimal visual outcomes for OPG, molecular therapy shows good potential (2, 35). Furthermore, avoidance of radiotherapy is also preferred wherever possible. The reassuring results of targeted therapy trials are helping to establish them as reasonable alternatives to radiation or previous chemotherapy regimens. The clinical benefit of targeted therapy appears to uphold previous management ideals with their promising effect on functional outcomes as much, if not more, than on tumor response. To deploy these therapies (**Figure 1**), however, tissue must be acquired surgically.

## Role of Biopsy for Optic Pathway Glioma

Historically, the role for biopsy was limited in the setting of OPGs. They are not amenable to complete resection, are histologically benign, involve critical neuroendocrine structures, and often respond favorably to chemotherapy. Based on these criteria, treatment paradigms de-emphasized the role of surgery. Existing consensus supports biopsy in cases that demonstrate atypical imaging findings in patients with NF1 (i.e., location outside the optic pathway, peripheral enhancement, areas of necrosis, diffusion restriction), or in sporadic OPG patients who are involved in relevant clinical trials (3) and concordant practice has been reported by multiple groups (5, 6, 55). While tissue sampling or tumor debulking can often be completed safely, it is important to recognize the risks of surgery for OPG, and discuss this with patients, families, and the multidisciplinary team prior to surgery. Post-operative complications can include visual deficits, endocrine dysfunction, hypothalamic disturbance, hemorrhage, and even, in more severe cases, death (3, 8). At our institution, to support the role and importance of knowing the genetic makeup of these tumors, we have evolved towards a more aggressive operative biopsy strategy, which includes tissue sampling for all patients with non-NF-1 associated OPGs, and for those with NF-1 who have particularly atypical imaging features or who demonstrate clinical or radiographic progression. In highly selected cases, operative debulking for tumors with significant extension into the third ventricle is undertaken.

In general, biopsy rates have increased in recent years, guiding the use of targeted therapies (5). There exists a further rationale that molecular profiling of all non-NF1 associated OPGs contributes to more effective management. Authors have discussed the fact that many of the common risks associated with operative biopsy can also eventually result from disease progression (11, 13). A proactive approach that uses operative biopsy to facilitate more effective tumor control may result in an improved overall risk/benefit ratio. In addition to improving decision making around medical anti-tumor regimens, an understanding of the biology of a given patients tumor may help identify therapies that defer or avoid the need for radiation. In the setting of research, one of the more significant limitations of modern clinical trials, such as PBTC-029 (NCT01089101), has been the lack of tissue sampling (29). As such, molecular characterization will have to be strongly considered in the context of late phase clinical trials.

## Surgical Approaches to Optic Pathway Glioma

If surgical resection is not indicated, options for tissue sampling include endoscopically assisted biopsy, stereotactic needle biopsy, and open biopsy. The optimal approach is dependent on tumor location and its relationship to important anatomic structures and should be determined on an individualized basis. Improvements in imaging quality and intraoperative navigation allow for precise operative planning and anatomical understanding, thereby improving the safety of surgical biopsy, regardless of the specific approach that is selected (8). Stereotactic needle biopsy can be planned pre-operatively using navigation software, which allows for the selection of a trajectory that avoids blood vessels and crucial neural structures. Surgical approaches for open biopsy include subfrontal eyebrow, pterional, interhemispheric transcallosal, interhemispheric trans-lamina terminalis, trans-foraminal (cortical), and endonasal trans-sphenoidal. Of note, it is important to consider that molecular analysis often demands a larger tissue sample than is necessary for a standard histopathological diagnosis. At our institution, to perform molecular studies, we acquire a specimen volume of at least five cubic millimeters, and specimen adequacy is verified intraoperatively. It is crucial that a team of neurosurgeons, neuro-oncologists, and pathologists work together closely to optimize the process of tissue acquisition and analysis.

## Additional Role for Surgery for Optic Pathway Glioma

While aggressive operative resection is not standard for most OPGs, neurosurgical intervention for patients with OPG in certain contexts is not controversial. The association between OPGs and hydrocephalus is well recognized and may require prompt surgical attention (5, 6, 12, 18). While most CSF diversion procedures in this context will be ventriculoperitoneal (VP) shunt placements, endoscopic third ventriculostomy (ETV) has been utilized in select cases (5). In the case of OPGs with significant extension into the third ventricle, restoration of normal CSF flow can be achieved through direct tumor debulking. This approach provides the added benefit of providing tissue for diagnosis and molecular studies. Cystic tumor components are commonly observed in OPGs, but can prove troublesome to manage (4). Medical therapies can contribute to cyst growth and sometimes multiple surgical procedures to decompress cysts are required. While simple cyst drainage is an option, Ommaya reservoir placement has also been utilized (5, 6).

An additional indication for surgical debulking is symptomatic mass effect (4–6, 18). When debulking is the operative goal, common approaches include pterional and interhemispheric transcallosal. The former is well-suited for laterally projecting tumors as well as for optic apparatus decompression. The midline interhemispheric approach may be preferred when the tumor debulking is focused on the third ventricle. This approach facilitates identification of a plane between the tumor and the ventricular wall, which decreases the risk of hypothalamic injury. Challenges associated with the interhemispheric approach include a relatively narrow operative corridor, risk of forniceal injury, and difficulty visualizing/accessing inferior tumor, which is close to optic pathway. This risk can be mitigated by limiting the goals of surgery to removal of tumor from the third ventricle, thereby discontinuing resection before areas of highest risk are encountered.

## Considerations for Low/Middle Income Countries

While we endorse the use of biopsy/surgery to obtain the most molecular data possible to help guide therapy, we recognize there may be limitations to the use of these methods in low/middle income countries (LMIC). The importance of subgroup directed therapy has been shown to be important and encouraged to be considered while treatment planning in countries with limited resources (56) and groups are in the process of developing lower-cost methods of assigning subgroups in different tumor types (57, 58). The European Society for Peadiatric Oncology (SIOP) Paediatric Oncology in Developing Countries working group has developed treatment guidelines for LGG diagnosed in LMIC based on service level abilities (59). This group also recommends biopsy for unresectable LGG if there are questions considered necessary to make a definitive diagnosis. Molecular targeted therapy is not addressed but would depend not only on the LMIC ability to provide reliable molecular testing (or obtain it through a secondary pathology consult), but also the ability to obtain the drugs which continue to carry a significant cost. If neither molecular testing or targeted drugs are financially available, chemotherapy and radiation recommendations are available tailored to the service level of different treatment centers (59).

## Conclusions

Optic pathway glioma can be associated with good outcomes regarding both tumor control and quality of life. The role of surgery for these patients is dynamic and exists on a spectrum that spans from clear indications to more controversial interventions. The promise and evolution of molecularly targeted treatments has increased the role of surgical intervention for these patients as tissue diagnosis is required for proper therapy selection. This management approach also demonstrates the need for well-established comprehensive multidisciplinary care in the treatment of optic pathway gliomas. Through utilization of a team of physicians that includes neuro-oncologists, neurosurgeons, ophthalmologists, pathologists, radiation oncologists, endocrinologists, and several other allied health professionals, optimal care of these patients throughout childhood will afford them the best chance of successful care.
